# Perturbed atrial calcium handling in an ovine model of heart failure: Potential roles for reductions in the L-type calcium current

**DOI:** 10.1016/j.yjmcc.2014.11.017

**Published:** 2015-02

**Authors:** Jessica D. Clarke, Jessica L. Caldwell, Margaux A. Horn, Elizabeth F. Bode, Mark A. Richards, Mark C.S. Hall, Helen K. Graham, Sarah J. Briston, David J. Greensmith, David A. Eisner, Katharine M. Dibb, Andrew W. Trafford

**Affiliations:** a Institute of Cardiovascular Science, Manchester Academic Health Science Centre, 3.24 Core Technology Facility, 46 Grafton St, Manchester M13 9PT, UK; b Liverpool Heart and Chest Hospital, Thomas Drive, Liverpool L14 3PE, UK

**Keywords:** Calcium, Atria, Heart failure, Sarcoplasmic reticulum

## Abstract

Heart failure (HF) is commonly associated with reduced cardiac output and an increased risk of atrial arrhythmias particularly during β-adrenergic stimulation. The aim of the present study was to determine how HF alters systolic Ca^2 +^ and the response to β-adrenergic (β-AR) stimulation in atrial myocytes. HF was induced in sheep by ventricular tachypacing and changes in intracellular Ca^2 +^ concentration studied in single left atrial myocytes under voltage and current clamp conditions. The following were all reduced in HF atrial myocytes; Ca^2 +^ transient amplitude (by 46% in current clamped and 28% in voltage clamped cells), SR dependent rate of Ca^2 +^ removal (*k*_SR_, by 32%), L-type Ca^2 +^ current density (by 36%) and action potential duration (APD_90_ by 22%). However, in HF SR Ca^2 +^ content was increased (by 19%) when measured under voltage-clamp stimulation. Inhibiting the L-type Ca^2 +^ current (*I*_Ca-L_) in control cells reproduced both the decrease in Ca^2 +^ transient amplitude and increase of SR Ca^2 +^ content observed in voltage-clamped HF cells. During β-AR stimulation Ca^2 +^ transient amplitude was the same in control and HF cells. However, *I*_Ca-L_ remained less in HF than control cells whilst SR Ca^2 +^ content was highest in HF cells during β-AR stimulation. The decrease in *I*_Ca-L_ that occurs in HF atrial myocytes appears to underpin the decreased Ca^2 +^ transient amplitude and increased SR Ca^2 +^ content observed in voltage-clamped cells.

## Highlights

• In an ovine model of heart failure systolic Ca^2 +^ is reduced in atrial myocytes.
• Action potential duration shortening is not responsible for changes in [Ca^2 +^]_i_.
• Reductions in L-type Ca^2 +^ current underpin the smaller systolic Ca^2 +^ transient.
• NCX current is reduced in heart failure but sarcolemmal Ca^2 +^ extrusion increased.
• Decreased Ca^2 +^ buffering power accelerates sarcolemmal Ca^2 +^ extrusion via NCX.

## 1 Introduction

Heart failure (HF) remains a leading cause of morbidity and mortality [1] as well as a major risk factor for the development of atrial fibrillation (AF) [2]. In the healthy heart atrial contraction increases ventricular filling and thus cardiac output by approximately 20% [3]. In HF however, the atrial contribution to ventricular filling is reduced and thus reduced atrial contractility contributes to the reduction in cardiac output observed in HF [4]. Whilst the cellular mechanisms leading to reduced contractility in the ventricle in HF have been extensively studied e.g. [5–9], the cellular changes that occur in the atria as result of the development of HF, and how these may contribute to atrial dysfunction in HF, are less well-understood.

Atrial remodelling as a result of HF could lead to reduced atrial contractility and an increased propensity for arrhythmias in at least three general ways; i) through changes to cellular Ca^2 +^ homeostasis reducing the amplitude of the systolic Ca^2 +^ transient or via facilitation of delayed after-depolarizations (DADs) [10], ii) via action potential shortening which could arise as a consequence of changes in the systolic Ca^2 +^ transient or ion channel remodelling (reviewed in [11]) and, iii) as a result of extracellular matrix remodelling leading to fibrosis, increased tissue stiffness and conduction block [12]. There are several reports that the atrial L-type Ca^2 +^ current (*I*_Ca-L_) is reduced in HF [13–16]. Given the importance of *I*_Ca-L_ in triggering Ca^2 +^ release from the sarcoplasmic reticulum (SR) [17,18], the reduction of atrial *I*_Ca-L_ is potentially significant as it will firstly reduce the amplitude of the systolic Ca^2 +^ transient and thus contractility as well as shortening action potential duration (APD) [19] and atrial effective refractory period thus potentially facilitating re-entrant arrhythmia formation [11].

Despite the extensive data showing that *I*_Ca-L_ is reduced in the atria in various disease settings, there remains a paucity of information regarding how cellular Ca^2 +^ handling is *quantitatively* altered in the atria in HF and the impact that changes in *I*_Ca-L_ have on SR Ca^2 +^ content and the systolic Ca^2 +^ transient. Using a brief period of rapid ventricular pacing in the dog, Yeh et al. [20] demonstrated larger Ca^2 +^ transients that were attributed to increased SR Ca^2 +^ loading (measured qualitatively) and a prolonged action potential duration in ‘HF’ myocytes; however, a role for changes in *I*_Ca-L_, or other Ca^2 +^ homeostatic mechanisms such as SERCA activity and Ca^2 +^ buffering [5,21] was not examined. Moreover, adrenergic stimulation plays a critical role in regulating cardiac contractility and the initiation of arrhythmias [22–24]. However, apart from limited studies on the effects of β-adrenergic (β-AR) stimulation on atrial *I*_Ca-L_ in cardiac disease states [16,25,26], how β-AR stimulation modulates systolic Ca^2 +^, SR Ca^2 +^ content and cellular Ca^2 +^ homeostasis in the atria during HF is unknown.

Thus, the objectives of the present study were to assess the impact of HF on atrial myocyte intracellular Ca^2 +^ homeostasis, systolic Ca^2 +^ and SR Ca^2 +^ content and how these are modified by β-AR stimulation. We used an ovine model of dilated cardiomyopathy produced by rapid ventricular pacing and found that HF leads to a shortening of the atrial action potential duration (APD) and reduction in Ca^2 +^ transient amplitude but SR Ca^2 +^ content, determined from the amplitude of the caffeine evoked rise of [Ca^2 +^]_i_, was unaltered. Under voltage clamp conditions the Ca^2 +^ transient remained smaller in HF and this occurred due to a reduction in *I*_Ca-L_ rather than a decrease in SR Ca^2 +^ content, which was paradoxically *increased*. During β-AR stimulation the Ca^2 +^ transient was increased more in HF than control cells. However, whilst SR dependent Ca^2 +^ removal and *I*_Ca-L_ were both augmented by β-AR stimulation in HF cells, they remained reduced compared to control cells. Interestingly, SR Ca^2 +^ content was highest in voltage-clamped HF atrial cells during β-AR stimulation. In action potential clamped myocytes the shortened HF APD does not explain the smaller systolic Ca^2 +^ transient. We therefore conclude that the decreased *I*_Ca-L_ in HF atrial myocytes is the major factor causing the smaller systolic Ca^2 +^ transient.

## 2 Methods

All procedures were in accordance with The United Kingdom Animals (Scientific Procedures) Act, 1986 and European Union Directive 2010/63. Institutional approval was received from The University of Manchester Animal Welfare and Ethical Review Board.

A detailed description of the experimental methods used in this study is available in the online supplement.

### 2.1 Experimental heart failure

HF was induced in 25 female Welsh sheep (35.2 ± 1.3 kg, ~ 18 months of age) by transvenous right ventricular tachypacing as described in detail previously [5,27,28]. For surgical procedures anaesthesia was induced and maintained by isoflurane inhalation (1–4% v/v) and perioperative analgesia (meloxicam, 0.5 mg/kg) and antibiosis (enrofloxacin, 2.5 mg/kg) were provided. Animal welfare and the clinical signs indicating the onset of HF were monitored at least once daily. On development of signs of HF (lethargy, dyspnoea and cachexia), ventricular tachypacing was stopped and para-sternal echocardiography was performed on conscious unsedated animals gently restrained in a sitting position. Animals were killed by intravenous administration of pentobarbitone (200 mg/kg) mixed with heparin (10,000 IU) to prevent coagulation in the coronary vasculature. Age and weight-matched animals were used as controls for the study. Animals were randomly assigned to the control or HF arms of the study and once enrolled, no animals were subsequently excluded from analyses.

### 2.2 Isolated myocyte studies

**Fig. 1:**
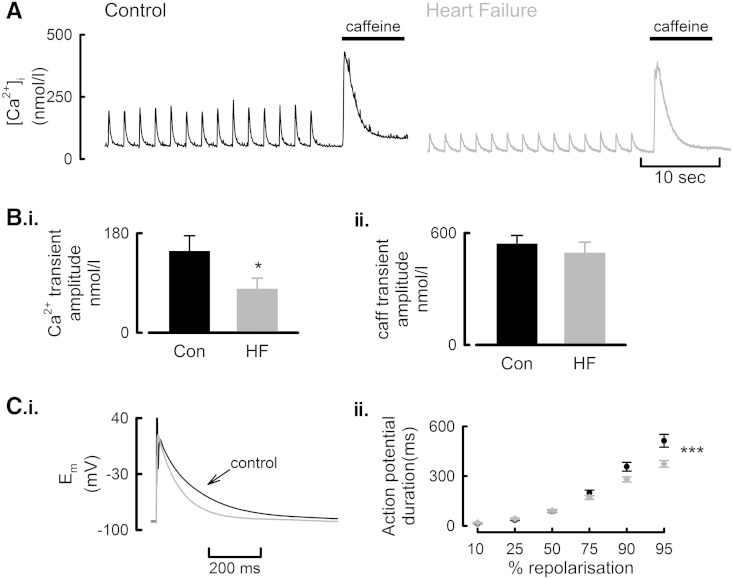
Action potential duration and Ca^2 +^ transient amplitude are reduced in HF atrial myocytes. A. Representative systolic and caffeine evoked Ca^2 +^ transients from Fura-2 loaded control (left) and HF (right) atrial myocytes following current clamp stimulation at 0.5 Hz, 37 °C. B. Mean data summarizing Ca^2 +^ transient amplitude (i) and, the amplitude of the caffeine evoked rise of [Ca^2 +^]_i_ (ii). C. Example action potentials from a control and HF cell (i) and, summary data for action potential repolarization times (ii). ^⁎^*P* < 0.05; ^⁎⁎⁎^*P* < 0.001.

**Fig. 2:**
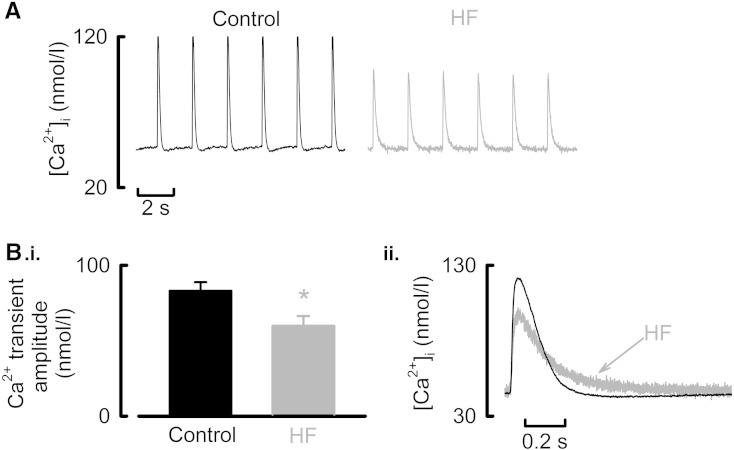
Reduced Ca^2 +^ transient amplitude and slowed relaxation in voltage-clamped HF atrial myocytes. A. Representative systolic Ca^2 +^ transients from a control (left) and HF atrial (right) myocyte. Cells were voltage-clamped, stimulated from a holding potential of − 40 mV with a 100 ms, 50 mV step at 0.5 Hz, 37 °C and changes in [Ca^2 +^]_i_ measured with Fluo-5F. B. (i) Mean data summarizing changes in Ca^2 +^ transient amplitude. (ii) Superimposed Ca^2 +^ transients from representative control and HF atrial cells demonstrating a slowed rate of decay of [Ca^2 +^]_i_. ^⁎^*P* < 0.05.

**Fig. 3:**
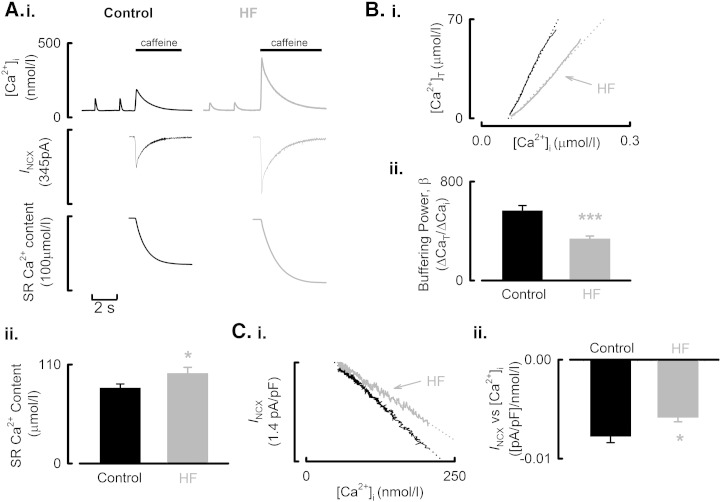
SR Ca^2 +^ content is increased and cellular Ca^2 +^ buffering power reduced in HF atria. A. (i) Quantification of SR Ca^2 +^ content in voltage-clamped control (left) and HF (right) atrial cells. Following steady state-stimulation membrane potential was held at − 40 mV and 10 mmol/l caffeine applied rapidly as indicated to discharge the SR store resulting in an inward NCX current (middle panels) which was integrated (lower panels) to quantify SR Ca^2 +^ content. (ii) Mean data for SR Ca^2 +^ content. B. (i) Representative Ca^2 +^ buffer slopes from the cell types indicated. Data obtained by plotting total Ca^2 +^ ([Ca^2 +^]_T_, ordinate) as a function of [Ca^2 +^]_i_ (abscissa). Total Ca^2 +^ and [Ca^2 +^]_i_ are both derived from caffeine evoked Ca^2 +^ transient. The broken lines fitted through the original data are best-fit linear regressions. (ii) Summary data showing mean data for cellular Ca^2 +^ buffering power (β). C. (i) Representative data showing the relationship between NCX current (*I*_NCX_) and [Ca^2 +^]_i_ obtained during the decay phase of the caffeine-evoked Ca^2 +^ transient. The broken lines through the original data are best-fit linear regressions. (ii). Mean data for the slope of the linear regression applied to the *I*_NCX_–[Ca^2 +^]_i_ relationship. ^⁎^*P* < 0.05; ^⁎⁎⁎^*P* < 0.001.

**Fig. 4:**
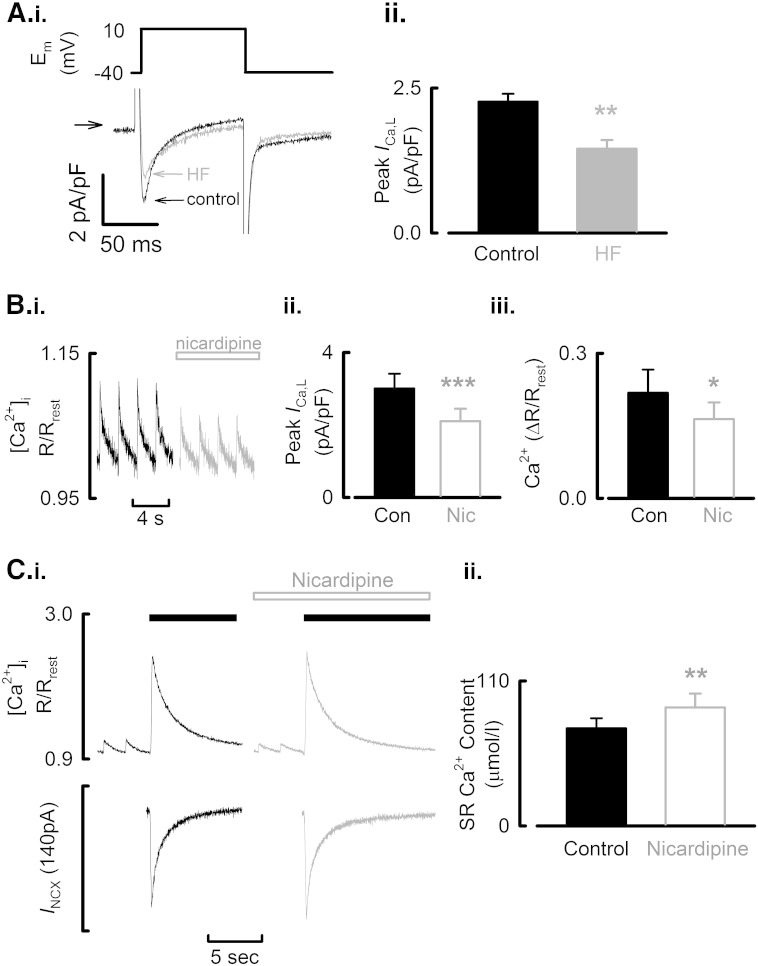
Decreased *I*_Ca-L_ in HF reduces systolic Ca^2 +^ and increases SR Ca^2 +^ content. A. (i) Representative *I*_Ca-L_ traces elicited by step depolarizations as indicated above the current record. (ii) Mean data summarizing peak *I*_Ca-L_ in control and HF atrial myocytes. B. (i) Experimental time course: the application of nicardipine (5 μmol/l) decreases the systolic Ca^2 +^ transient. [Ca^2 +^]_i_ measured using Fura-2. Data obtained on different experimental apparatus and expressed as pseudo-ratio (R/R_rest_) relative to the resting ratio of emitted light excited at 340 nm and 380 nm. (ii) Mean data summarizing the effect of nicardipine on peak *I*_Ca-L_. (iii) Mean data summarizing the effect of nicardipine on Ca^2 +^ transient amplitude. C. (i) The effect of nicardipine on SR Ca^2 +^ content quantified by the rapid application of caffeine (10 mmol/l, solid bars) to a control cell following steady-state stimulation in the absence (left) and presence (right) of nicardipine (5 μmol/l, open bar). (ii) Mean data summarizing the effect of nicardipine on SR Ca^2 +^ content. ^⁎^*P* < 0.05; ^⁎⁎^*P* < 0.01; ^⁎⁎⁎^*P* < 0.001.

**Fig. 5:**
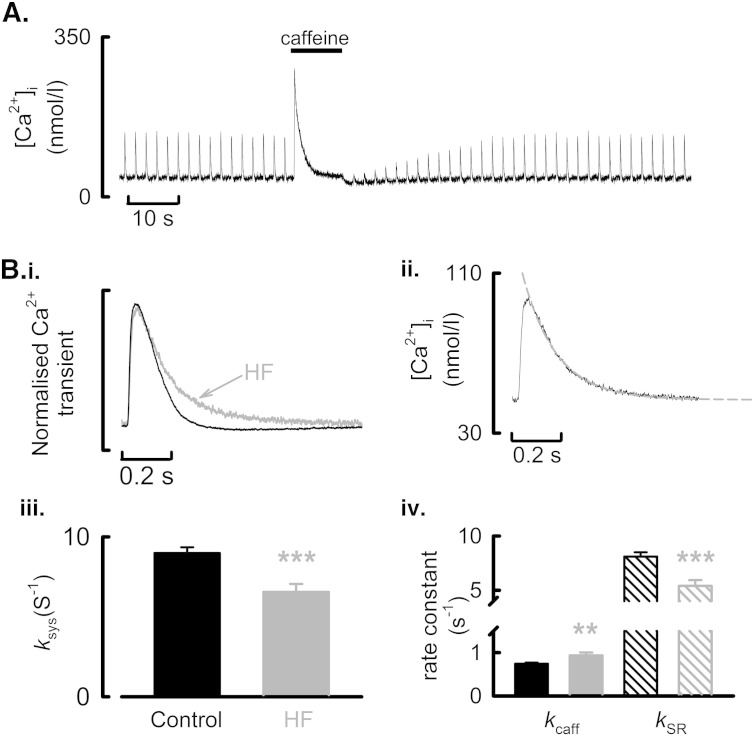
Slowed rates of decay of systolic Ca^2 +^, reduced SR mediated Ca^2 +^ uptake and enhanced sarcolemmal Ca^2 +^ extrusion in HF atrial myocytes. A. Experimental time course illustrating how SR and non-SR dependent rates of Ca^2 +^ removal are calculated in voltage-clamped atrial myocytes. Following steady-state stimulation caffeine is applied to discharge the SR store. B. (i) Normalized systolic Ca^2 +^ transients from a representative control and HF atrial cell as indicated. (ii) Method for determining the rate of decay of the systolic Ca^2 +^ transient (*k*_sys_) by fitting (broken line) a single exponential function to the data. (iii) Summary data for the calculated rates of decay of the systolic Ca^2 +^ transient. (iv) Summary data for rate constants of decay of [Ca^2 +^]_i_ due to sarcolemmal extrusion (*k*_caff_) and SR mediated uptake (*k*_sys_). ^⁎⁎^*P* < 0.01; ^⁎⁎⁎^*P* < 0.001.

All experiments (except cell volume measurements, Supplemental Fig. S.I) were performed at 37 °C. Changes in intracellular Ca^2 +^ concentration ([Ca^2 +^]_i_) were monitored using the acetoxymethyl ester of either Fluo-5F (5 μM; Figs. 2, 3 & 5) or Fura-2 (5 μM; Figs. 1, 4 & 7). Where Fluo-5F was used to measure [Ca^2 +^]_i_ we were unable to reliably obtain Ca^2 +^ saturated fluorescence values and therefore changes in [Ca^2 +^]_i_ were calibrated by determining the resting [Ca^2 +^]_i_ in Fura-2 loaded cells [29] (following in vitro calibration of the experimental system; Molecular Probes, Invitrogen UK), and then calibrated as originally described by Cheng et al. [30] assuming a *K*_d_ for Ca^2 +^ of 1035 nmol/l [31]. Single atrial myocytes were current or voltage-clamped using the perforated patch technique with amphotericin-B (240 μg/ml) [7,32,33]. The surface area to volume ratio (Supplementary data Fig. S.I) was calculated in calcein-AM (20 μmol/l) loaded cells as described previously [32].

SR Ca^2 +^ content was calculated by the rapid application of 10 mM caffeine and integration of the resulting Na^+^–Ca^2 +^ exchange (NCX) current [7,32–34]. The Ca^2 +^ buffering capacity of cells was also obtained from the caffeine evoked Ca^2 +^ transients [21,32,35].

### 2.3 Immunoblotting

Left atrial tissue was snap frozen and stored in liquid N_2_ until use. Samples were prepared in RIPA buffer and subject to denaturing SDS-PAGE as described previously [5,32,36]. The expression of the so-called ‘house-keeping’ proteins β-actin and GAPDH were altered in this model of HF (Supplementary data Fig. S.II). We therefore used an internal standard on all blots. This internal standard was obtained from a single control animal and used to normalize protein expression on each gel [5,28]. Three replicate gels were obtained and data averaged for each sample. Details of protein loading, antibody concentrations and sources are provided in the online supplementary information.

### 2.4 Statistics

Initial estimates of sample sizes were determined by power analysis using Sigmaplot 11 (Systat Software Inc., USA) assuming α = 0.05 and β = 0.8 and were based on the level of effects and standard deviations observed previously in this model [5,27,28,37]. Normality of data distribution was assessed using the Kolmogorov–Smirnov statistic (IBM SPSS Statistics v20). Data are expressed either as mean ± standard error of the mean (SEM) for normally distributed data or as the median (inter-quartile range) for non-normally distributed data from *n* cells and *N* animals (e.g. *n*/*N* control; *n*/*N* HF). Continuous scale data was log_10_ transformed [38] and differences between control and HF animals determined using linear mixed modelling to account for instances where multiple cellular observations (*n*) were obtained from each experimental subject (*N*) (IBM SPSS Statistics v20). Where treatments/effects were within the same animal, cellular differences were assessed using a paired Students t-test and considered significant when *P* < 0.05.

## 3 Results

### 3.1 Cardiac and cellular remodelling in heart failure

Clinical signs of HF (lethargy and dyspnoea) were present after a median 33.5 (29.8–37) days of tachypacing. Due to the anatomic orientation of the cardiac apex over the sternum in sheep we were unable to obtain trans-thoracic four-chamber views and thus measures of atrial dimensions or E/A ratios. However ventricular dilatation was present as we have found previously [5]. In the animals used in the present study left ventricular internal diastolic dimension increased by 47.2 ± 8.3% and fractional shortening decreased by 57.1 ± 1.9% (both *P* < 0.001). Atrial cellular hypertrophy was noted in isolated left atrial myocytes (Supplementary Fig. SI.A) with an increase in planar width from 15.0 ± 0.2 to 18.1 ± 0.4 μm and length from 127.5 ± 1.7 to 168.5 ± 3.5 μm (both *P* < 0.001; 347/23 control; 110/9 HF). Membrane capacitance also increased from 59.6 ± 2.1 to 125.9 ± 9.3 pF (Supplementary Fig. SI.B, *P* < 0.001). From simultaneously derived confocal 3-dimensional rendered cell volumes and membrane capacitance measurements [32,39], the depth of the cells increased in HF (control, 9.5 ± 0.4 μm; HF 16.5 ± 1.1 μm, *P* < 0.001. 19/5 control; 15/5 HF). However, the calculated surface area to volume ratio was unaltered (control, 5.06 ± 0.17 pF/pl; HF, 4.89 ± 0.26 pF/pl. *P* = 0.58). The respective surface area to volume ratio was used to express all integrated cellular Ca^2 +^ fluxes and SR Ca^2 +^ contents to *total* cellular volume [7,33].

### 3.2 Decreased atrial Ca^2 +^ transient amplitude and action potential duration in heart failure

We first sought to determine if the cellular hypertrophy and chamber dilatation were associated with alterations to the systolic Ca^2 +^ transient. Under current clamp conditions, action potential evoked systolic Ca^2 +^ transients were elicited at 0.5 Hz and representative traces shown in Fig. 1A. Diastolic [Ca^2 +^]_i_ was reduced in current-clamped HF myocytes control, 60.7 ± 4.5; HF, 37.3 ± 4.8 nmol/l, *P* = 0.001. However, the amplitude of the action potential evoked rise of systolic Ca^2 +^ was reduced in HF atrial myocytes by 46 ± 17% (Fig. 1B.i, *P* < 0.05; 18/6 control; 16/5 HF). Conversely, despite the smaller systolic rise of [Ca^2 +^]_i_, the amplitude of the caffeine evoked rise of [Ca^2 +^]_i_, used as a qualitative measure of SR Ca^2 +^ content, was unaltered following current clamp stimulation (Fig. 1B.ii). The reduction in systolic Ca^2 +^ was however associated with a shortening of the action potential duration in HF atrial myocytes (Fig. 1C, *P* < 0.001) with the time to 90% repolarization decreasing from 357 ± 26 ms in control to 280 ± 15 ms in HF.

We then sought to determine the potential mechanisms by which the systolic Ca^2 +^ transient amplitude was reduced in HF atrial myocytes. In the first series of experiments the action potential clamp technique was used to apply the averaged control and HF action potential waveforms to myocytes isolated from control atria (Supplementary material, Fig. S.III). Following stimulation with the HF atrial action potential there was no detectable change in the amplitude of the systolic Ca^2 +^ transient or caffeine-evoked rise of [Ca^2 +^]_i_ or SR Ca^2 +^ content measured from the integral of the NCX current following caffeine application (Supplementary data, Fig. S.III). Thus, changes in action potential duration do not explain the smaller atrial systolic Ca^2 +^ transients observed in this model of HF.

We then performed voltage clamp experiments to facilitate quantitative analysis of *I*_Ca-L_, cellular Ca^2 +^ fluxes and SR Ca^2 +^ content. Representative Ca^2 +^ transients following steady-state voltage clamp stimulation are illustrated in Fig. 2A. On average the amplitude of the atrial systolic Ca^2 +^ transient was reduced by 28 ± 9% (Fig. 2B.i, *P* < 0.05; 43/16 control; 21/10 HF). Given the strong dependence of Ca^2 +^ transient amplitude on SR Ca^2 +^ content and the indication, from the slowed decay of [Ca^2 +^]_i_ in HF (Fig. 2B.ii) that SERCA activity is impaired, we next investigated if the decrease of Ca^2 +^ transient amplitude was due to a fall of SR Ca^2 +^ content. Following steady-state stimulation, caffeine (10 mmol/l) was applied rapidly to the cell and the resulting NCX current integrated. This data is summarized in Fig. 3A. In HF atrial myocytes SR Ca^2 +^ content was *increased* from 83.9 ± 4.5 to 100.1 ± 6.7 μmol/l (*P* < 0.05; 24/8 control; 17/10 HF). Therefore, the reduced Ca^2 +^ transient amplitude observed in HF atrial cells cannot be explained by a decrease of SR Ca^2 +^ content.

Another possible explanation for the smaller systolic Ca^2 +^ transients observed in HF atrial cells would be an increase in the Ca^2 +^ buffering power of the cells. Representative Ca^2 +^ buffering curves are illustrated in Fig. 3B.i. The relationship between *total* and *free* Ca^2 +^ is shallower in the HF atrial cells (Fig. 3B.ii, control, 565 ± 35; HF, 318 ± 42). On average the Ca^2 +^ buffering power of the cells (ratio of *total* to *free* Ca^2 +^) was *reduced* by 43.7 ± 8.2% in HF (*P* < 0.001; 24/9 control; 17/10). Therefore, as with SR Ca^2 +^ content, changes in Ca^2 +^ buffering power do not explain the smaller systolic Ca^2 +^ transient in HF atrial cells.

The dependence of NCX on [Ca^2 +^]_i_ was also assessed from the caffeine evoked rise of [Ca^2 +^]_i_. The representative traces and summary data (Fig. 3C) show that less NCX current is produced for a given change in [Ca^2 +^]_i_ in HF atrial myocytes indicating a down-regulation of NCX activity in HF. On average the slope of the relationship between *I*_NCX_ and [Ca^2 +^]_i_ was decreased by 24.9 ± 8.7% (Fig. 3C.ii, *P* < 0.05; 24/9 control; 15/9 heart failure).

### 3.3 Decreased L-type Ca^2 +^ current and role in altered systolic Ca^2 +^ and SR Ca^2 +^ content in heart failure

Given that the increase of SR Ca^2 +^ content and reduction in Ca^2 +^ buffering power in HF atrial cells are unable to account for the decreased Ca^2 +^ transient amplitude observed in HF atrial myocytes, we next investigated a potential role for changes in the L-type Ca^2 +^ current (*I*_Ca-L_) as this has both trigger and loading roles. The representative *I*_Ca-L_ records and summary data of Fig. 4A show that HF was associated with a reduction in the peak *I*_Ca-L_ density (by 36.1 ± 7.8%; control, 2.27 ± 0.14 pA/pF; HF, 1.45 ± 0.15 pA/pF. *P* < 0.005; 47/20 control; 21/10 heart failure). However, the amount of Ca^2 +^ entry via the L-type Ca^2 +^ current calculated by integrating *I*_Ca-L_ was unchanged in HF (control, 0.74 ± 0.06 μmol/l; HF, 0.86 ± 0.09 μmol/l. *P* = 0.827). The unchanged integrated Ca^2 +^ entry is associated with a slowed time course of inactivation of *I*_Ca-L_ in HF (control, 404 ± 50 s^− 1^; HF, 171 ± 23 s^− 1^, *P* < 0.01).

The role that the decreased *I*_Ca-L_ has in producing the smaller Ca^2 +^ transient in HF atrial cells was then investigated using the approach illustrated in Fig. 4B. In control cells, nicardipine (5 μmol/l) was applied to reduce *I*_Ca-L_ to a similar extent to that observed in HF (Fig. 4B.ii, by 30 ± 15%). This reduction of *I*_Ca-L_ caused a 27 ± 15% decrease in Ca^2 +^ transient amplitude (Fig. 4B.iii, *P* < 0.01). However, unlike the maintained integrated Ca^2 +^ entry via *I*_Ca-L_ in HF atrial myocytes, the integrated Ca^2 +^ entry in response to nicardipine was reduced (from 0.9 ± 0.2 to 0.7 ± 0.2 μmol/l; *P* < 0.01; 8 cells from 4 control hearts). The reduced integrated Ca^2 +^ entry presumably reflects the combined effects of the decreased peak and unaltered rate of inactivation of the current in nicardipine. A potential role for altered L-type Ca^2 +^ channel single channel kinetics in HF, specifically increased open probability and availability [40], would also contribute to the differential effects on *I*_Ca-L_ inactivation in HF and nicardipine treated myocytes. Furthermore, the favoured mode 0 gating of the channel induced by the dihydropyridine antagonists [41] would also lead to the differential effects on *I*_Ca-L_ inactivation between HF and nicardipine treated cells. The effect that the pharmacological reduction of *I*_Ca-L_ had on SR Ca^2 +^ content is shown in Fig. 4C. The amplitude of the caffeine evoked rise of [Ca^2 +^]_i_ and inward NCX current are increased following nicardipine exposure (Fig. 4C.i) and this resulted in an increase of SR Ca^2 +^ content from 74.1 ± 7.6 μmol/l in control to 89.9 ± 10.5 μmol/l in nicardipine (Fig. 4C.ii, *P* < 0.01). This data therefore suggests that it is the reduction of *I*_Ca-L_ in HF atrial cells that is responsible for both the decrease of Ca^2 +^ transient amplitude and increase of SR Ca^2 +^ content.

Whilst the decrease in *I*_Ca-L_ therefore explains the smaller Ca^2 +^ transients and increased SR Ca^2 +^ content observed in voltage-clamped cells we also sought to determine if changes in SERCA or NCX activity also occur in HF and thus may contribute to the observed changes in systolic Ca^2 +^. Firstly we determined if SERCA activity was altered in HF using the approaches outlined in Fig. 5A. Normalized steady-state systolic Ca^2 +^ transients from a control and HF atrial cell are shown in Fig. 5B.i where it is clear that the systolic Ca^2 +^ transient decays more slowly in HF. This was quantified by fitting a single exponential to the decay phase of the Ca^2 +^ transient to obtain the rate constant of decay of systolic Ca^2 +^ (*k*_sys_, Fig. 5B.ii). On average, *k*_sys_ was reduced by 27.1 ± 6.3% in HF atrial cells (Fig. 5B.iii, Control, 8.99 ± 0.36 s^− 1^; HF, 6.55 ± 0.5 s^− 1^, *P* < 0.001; 43/18 control; 21/10 HF). The underlying mechanism for the reduction of *k*_sys_ in HF atrial cells was then determined by also fitting the decay phase of the caffeine evoked Ca^2 +^ transient with a single exponential (*k*_caff_). The SR dependent rate of Ca^2 +^ removal (*k*_SR_) was then derived by subtraction (*k*_SR_ = *k*_sys_ − *k*_caff_). In HF, sarcolemmal dependent Ca^2 +^ extrusion by NCX and PMCA (*k*_caff_) was increased by 25.2 ± 10% (control, 0.745 ± 0.024 s^− 1^; HF, 0.933 ± 0.069 s^− 1^; *P* < 0.01). Conversely, the SR dependent rate of Ca^2 +^ removal (*k*_SR_) was decreased (Fig. 5B.iv, Control, 8.1 ± 0.4 s^− 1^; HF, 5.4 ± 0.5 s^− 1^, *P* < 0.001). Thus the increase in surface membrane-mediated Ca^2 +^ extrusion (*k*_caff_) is inconsistent with the decrease in the slope of the relationship between *I*_NCX_ and [Ca^2 +^]_i_ (Fig. 3C). There are at least two possible explanations for this discrepancy; i) an increase in plasmalemmal Ca^2 +^-ATPase mediated Ca^2 +^ extrusion in HF and, ii) an effect of reduced cellular Ca^2 +^ buffering power (Fig. 3B). The effect of the reduced Ca^2 +^ buffering power is examined in Supplementary Fig. S.IV. We find that during the systolic Ca^2 +^ transient, in representative cells, Ca_T_ falls more slowly in the HF cell whereas Ca_T_ falls more quickly in the HF cell during the caffeine-evoked Ca^2 +^ transient. In Fig. S.IV.B, the relationship between *k*_caff_ and cellular Ca^2 +^ buffering power is examined in control and HF cells. An inverse correlation (Pearsons correlation coefficient, *P* = 0.01) exists indicating that as Ca^2 +^ buffering power decreases then *k_caff_* increases. We have also determined, by blocking NCX with 10 mmol/l Ni^2 +^ and measuring the rate of decay of the caffeine evoked rise of [Ca^2 +^]_i_, that the contribution of PMCA to Ca^2 +^ extrusion is no different between control and HF atrial myocytes (*k*_PMCA_: control, 1.17 ± 0.04 s^− 1^; HF, 1.09 ± 0.01 s^− 1^). The unaltered *k*_PMCA_, despite reduced Ca^2 +^ buffering, is further evidence that the decrease in Ca^2 +^ buffering power in HF accounts for the observed increase in *k*_caff_.

### 3.4 Molecular correlates of decreased SERCA function and reduced Ca^2 +^ transient amplitude

**Fig. 6:**
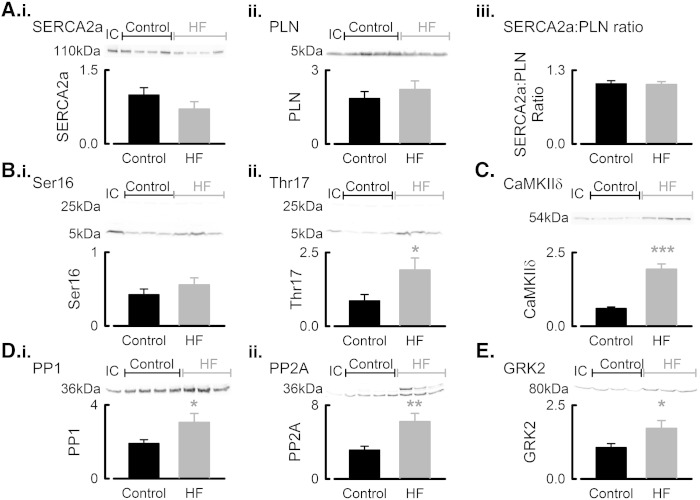
Molecular alterations impacting on atrial cellular Ca^2 +^ handling in HF. A. Representative Western blots (upper panel) and summary data (lower panel) for (i) SERCA2a, (ii) PLN and, (iii) summary of the SERCA2a:PLN. B. Representative Western blots and summary data for (i) Ser16 and (ii) Thr17 phosphorylated PLN. C. Determination of CAMKIIδ expression in heart failure atria showing Western blot (upper panel) and summary data (lower panel). D. Representative Western blots (upper panel) and summary data (lower panel) for (i) PP1 and, (ii) PP2a expression in HF. E. Increased GRK-2 expression in HF showing representative Western blot (upper panel) and summary data (lower panel). ^⁎^*P* < 0.05; ^⁎⁎^*P* < 0.01; ^⁎⁎⁎^*P* < 0.001.

The next experiments were designed to determine if changes in the expression and/or phosphorylation status of various proteins involved in controlling SERCA activity explain the reduced SERCA function noted in Fig. 5. In each case we used either 6 or 7 hearts from control and HF animals for biochemical analyes. There was no change in SERCA protein expression, total PLN expression, the SERCA to PLN expression ratio (Fig. 6A) or CSQ expression (Supplementary data, Fig. S.V). Using phospho-specific antibodies we also examined the phosphorylation status of PLN (Fig. 6B) and observed that whilst the phosphorylation status of the PKA-dependent residue (Ser-16) was unaltered in HF, there was increased phosphorylation at the adjacent Ca^2 +^-calmodulin kinase II- (CAMKII) dependent Thr-17 residue (to 221 ± 73% of control, *P* < 0.05); an effect most likely due to the 320 ± 40% increase (relative to control) in CAMKII expression in HF (Fig. 6C, *P* < 0.001). The unaltered PKA-dependent phosphorylation of PLN was associated with an increase in protein phosphatase 1 (PP1; to 159 ± 31%; *P* < 0.05; Fig. 6D.i), protein phosphatase 2a (PP2a, to 198 ± 38%; *P* < 0.01; Fig. 6D.ii) and G-protein receptor kinase 2 (GRK-2; to 161 ± 32%; *P* < 0.05; Fig. 6E) expression HF.

We then examined additional molecular correlates of the reduced Ca^2 +^ transient amplitude observed in HF atria and investigated RyR expression and phosphorylation at PKA- and CAMKII-dependent sites (Supplementary data; Fig. S.V). No differences in RyR expression, PKA-dependent Ser-2808 RyR phosphorylation or CAMKII-dependent Thr-2814 RyR phosphorylation were observed between control and HF atrial tissues.

### 3.5 Restoration of the systolic Ca^2 +^ transient in HF atrial cells by β-adrenergic stimulation; an effect via the L-type Ca^2 +^ current and SR Ca^2 +^ content

**Fig. 7:**
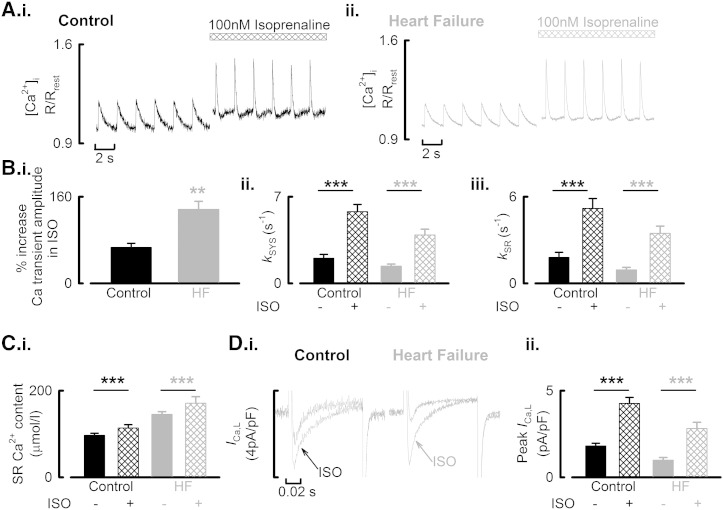
Normalization of the systolic Ca^2 +^ transient and impact of β-AR stimulation on cellular Ca^2 +^ homeostasis in HF atrial myocytes. A. Representative experimental time course from representative control (i) and HF (ii) cells. Cells were voltage-clamped and stimulated in the presence of 100 nmol/l isoprenaline as indicated above the records. Panels show steady-state effects. [Ca^2 +^]_i_ measured with Fura-2. Data obtained on different experimental apparatus and expressed as a pseudo-ratio (R/R_rest_) relative to the resting ratio of emitted light excited at 340 nm and 380 nm. B. (i) Isoprenaline mediated increase in Ca^2 +^ transient amplitude. (ii) Summary data for effect of β-AR stimulation on the rate of decay of the systolic Ca^2 +^ transient (*k*_sys_). (iii) Summary data for effect of β-AR stimulation on the SR dependent rate of Ca^2 +^ removal (*k*_SR_). C. Summary data for increase in SR Ca^2 +^ content during β-AR stimulation. D. (i) Representative *I*_Ca-L_ records from control and HF atrial myocytes under basal stimulation and following β-AR stimulation as indicated. (ii) Mean data summarizing the effect of β-AR stimulation on peak *I*_Ca-L_ density. ^⁎⁎^*P* < 0.01; ^⁎⁎⁎^*P* < 0.001.

To determine if the reduced Ca^2 +^ transient observed in HF atrial cells could be ‘rescued’, we examined the effects of the non-specific β-AR agonist isoprenaline (100 nmol/l) on Ca^2 +^ transient amplitude (Fig. 7A.i & ii). Isoprenaline increased Ca^2 +^ transient amplitude in both control and HF atrial cells although to a greater extent in HF cells (Fig. 7B.i; control, by 66 ± 8%; HF, by 137 ± 15%; *P* < 0.001; 24/11 control; 23/5 HF) such that during β-AR stimulation, Ca^2 +^ transient amplitude in HF was indistinguishable from that in control (not shown). Isoprenaline also accelerated the rate of decay of the systolic Ca^2 +^ transient in both control and HF atrial cells (*k*_sys_, Fig 7B.ii); an effect attributable to an increase in SERCA activity with *k*_SR_ increasing from 1.80 ± 0.36 s^− 1^ to 5.19 ± 0.68 s^− 1^ in control cells (*P* < 0.001) and, 0.93 ± 0.17 s^− 1^ to 3.47 ± 0.50 s^− 1^ in HF atrial cells (Fig. 7B.iii, *P* < 0.001). Again, during β-AR stimulation HF and control cells were statistically indistinguishable (*k*_sys_, *P* = 0.145; *k*_SR_, *P* = 0.187). The increase in SERCA activity during β-AR stimulation also increased SR Ca^2 +^ content in both control (97.0 ± 4.7 to 113.8 ± 7.6 μmol/l, *P* < 0.001) and HF (145.2 ± 6.4 to 171.1 ± 15.1 μmol/l, *P* < 0.01) atrial cells (Fig. 7C). However, during β-AR stimulation, SR Ca^2 +^ content was greatest in HF atrial cells (*P* < 0.001). The isoprenaline-mediated augmentation of Ca^2 +^ transient amplitude was also associated with an increase of L-type Ca^2 +^ current (Fig 7D.i) in both cell types (Fig. 7D.ii, control, 4.26 ± 0.35 pA/pF; HF, 2.81 ± 0.36 pA/pF; *P* < 0.001) although peak *I*_Ca-L_ was smaller in HF cells than in control cells (*P* < 0.05). In ISO the integrated Ca^2 +^ entry on *I*_Ca-L_ was indistinguishable between control and HF mycoytes. Finally, by Western blotting we were unable to detect differences in the expression of the G-proteins G_αs_ and G_αi(1/2/3)_ (Supplementary data; Fig S.VI).

### 3.6 The effects of HF-induced changes to intracellular Ca^2 +^ homeostasis on cellular Ca^2 +^ economy in the atria

The previous sections highlight multiple changes in cellular Ca^2 +^ homeostasis in atrial cells occurring in HF. How these impact on the total Ca^2 +^ economy of the cell are summarized in Table 1. The systolic Ca^2 +^ transient decreases by 28 ± 9% in HF. However, as a consequence of the decreased Ca^2 +^ buffering power (β) of the cell in HF the *total* Ca^2 +^ requirement to generate the systolic Ca^2 +^ transient is decreased by 59 ± 7% (*P* < 0.001). This *total* Ca^2 +^ requirement is met from two sources; i) Ca^2 +^ entry via *I*_Ca-L_ and, ii) the release of Ca^2 +^ from the SR. Given that the integrated Ca^2 +^ entry via *I*_Ca-L_ is unaltered and SR Ca^2 +^ content is *increased* in HF, it is not surprising therefore that the fractional release of Ca^2 +^ from the SR is reduced in HF by 64 ± 7% (*P* < 0.001). Finally, the gain of excitation contraction coupling can be calculated from the integrated Ca^2 +^ entry and release of Ca^2 +^ from the SR during the systolic Ca^2 +^ transient is reduced by 67 ± 7% (*P* < 0.001) in HF.

## 4 Discussion

The main findings from the present work are that in failing hearts atrial myocytes, i) are hypertrophied, ii) have smaller systolic Ca^2 +^ transients that decay more slowly, iii) have a reduction in *I*_Ca,L_, iv) have a reduced Ca^2 +^ buffering power and, iv) have a greater increase in Ca^2 +^ transient amplitude in response to β-AR stimulation. The reduction of *I*_Ca-L_ is sufficient to explain the smaller systolic Ca^2 +^ transient and the accompanying increased SR Ca^2 +^ content observed in voltage clamped cells. The reduced Ca^2 +^ buffering capacity of HF atrial myocytes is insufficient to maintain systolic Ca^2 +^, but it does facilitate the enhanced sarcolemmal-mediated rate of Ca^2 +^ extrusion observed in HF atrial myocytes. The restoration of systolic Ca^2 +^ during β-AR stimulation likely occurs as a result of increases in *I*_Ca-L_ and SR Ca^2 +^ content. The above changes in cellular Ca^2 +^ homeostasis provide a mechanism for the reduction in Ca^2 +^ transient amplitude observed in atrial myocytes isolated from failing hearts.

### 4.1 Cellular remodelling in heart failure

Two major considerations in the present manuscript are how HF induced by right ventricular tachypacing mediates changes in cellular geometry and atrial cellular Ca^2 +^ homeostasis. As such it is important to note (Supplementary material, Fig S.VII) that the high ventricular pacing rate used to initiate HF does not increase atrial rate. The changes in Ca^2 +^ homeostasis and cellular remodelling are therefore not due to atrial tachypacing in contrast to those observed in various models of atrial fibrillation e.g. [42,43]. In order to quantify the HF-induced changes in cellular Ca^2 +^ cycling, a measure of the surface area to volume ratio is required to relate sarcolemmal Ca^2 +^ fluxes and SR Ca^2 +^ content to the volume of the cell. In this model of HF, atrial myocytes are markedly hypertrophied showing a ~ 20% increase in cell diameter, ~ 30% increase in cell length and ~ 70% increase in cell depth. Both the increase of cell dimensions and loss of t-tubules that occurs in atrial cells in this HF model [27] would be expected to decrease the surface area to volume ratio. However, such a decrease is not observed in experiments where cell volume is derived from confocal z-stacks of calcein loaded cells patch-clamped to obtain a simultaneous measure of cell volume and membrane capacitance (surface area). Indeed, the HF and control cell data fit on precisely the same surface area (capacitance) to volume relationship even though their surface area and volume values overlap minimally. There are at least two potential factors that could explain the apparent constancy of the surface area to volume ratio in HF despite the cellular hypertrophy and loss of t-tubules [27]; i) the depth of the cells increased fractionally more than cell width consequently changing the expected shift in surface area to volume ratio (which is ‘based’ on the assumption that cells are ‘cylindrical’) and, ii) atrial dilatation may have stretched the cells and incorporated sub-membrane caveolae into the surface membrane and thus increased the apparent surface area to volume ratio. Sub-membrane caveolar incorporation has been suggested previously in acutely stretched myocytes [44]. Such effects would result in an increase in the observed surface area to volume ratio over that predicted from the changes in cellular planar dimensions.

### 4.2 Decreased *I*_Ca-L_ can explain the smaller systolic Ca^2 +^ transient and paradoxical increase of SR Ca^2 +^ content in voltage-clamped HF atrial cells

We find that a decrease of *I*_Ca-L_ accompanied the observed decrease of the systolic Ca^2 +^ transient. Such a decrease of atrial *I*_Ca-L_ has been observed previously in various models of cardiac disease e.g. [42,43,45,46]. That the decrease in *I*_Ca-L_ is *causal* of the decreased Ca^2 +^ transient is demonstrated by the observation that pharmacologically inhibiting *I*_Ca-L_ in control cells with nicardipine reproduces the effect of HF on *I*_Ca-L_, Ca^2 +^ transient amplitude *and* SR Ca^2 +^ content. Whilst we do not have data for changes in dihydropyridine binding or L-type Ca^2 +^ channel α-subunit expression in the atria in HF, the loss of atrial t-tubules that occurs in this model of HF [27] is likely to contribute to the fall in *I*_Ca-L_ [47].

The decrease of *I*_Ca-L_ may also explain the observed increase of SR Ca^2 +^ content in voltage-clamped myocytes. The L-type Ca^2 +^ current has two effects on SR Ca^2 +^; i) it triggers release and therefore an increase of *I*_Ca-L_ would be expected to decrease SR Ca^2 +^ and, ii) on the other hand it loads the cell and therefore the SR with Ca^2 +^. It is therefore difficult to predict the net effect of change in *I*_Ca-L_ on SR Ca^2 +^ content. We find that in the atria, as in the ventricle [5,18,48], reducing *I*_Ca-L_ leads to an increase of SR Ca^2 +^ content. However, the increase in SR Ca^2 +^ content is insufficient to restore the systolic Ca^2 +^ transient which remains smaller in HF atrial myocytes despite the increase in SR Ca^2 +^ content.

### 4.3 Altered cellular Ca^2 +^ buffering and t-tubule loss in heart failure

Whilst the smaller systolic Ca^2 +^ transient observed in HF atrial cells can be mimicked in control cells by inhibition of *I*_Ca-L_ with nicardipine, additional factors regulating the systolic Ca^2 +^ transient also change in HF. These include, i) reductions in the Ca^2 +^ buffering capacity and, ii) a decrease in the gain of excitation contraction coupling. The net effect of these changes however remains a smaller rise of Ca^2 +^ in HF atrial cells. A key factor likely contributing to the reduction in excitation contraction coupling gain is the reduction in peak (trigger) *I*_Ca-L_ in HF. Again, the loss of t-tubules in HF atrial myoyctes [27,42,49] and hence reduction in *I*_Ca-L_ [47,50] is therefore likely an important factor in the decrease in Ca^2 +^ transient amplitude we observe in HF atrial cells in the sheep.

The finding in the present study that, despite the reported loss of t-tubules in HF atrial myocytes [27], the relationship between NCX current and [Ca^2 +^]_i_ is reduced in HF is consistent with either a reduction of NCX expression at the cell surface in HF or, if this is unaltered, an increased density of NCX on the t-tubules which have subsequently been lost in HF. Given the importance of NCX in arrhythmogenesis via Ca^2 +^ dependent mechanisms we would suggest that, on balance, the decrease in NCX current we see in this model of end-stage heart failure might mean that other mechanisms e.g. as a result of action potential shortening or K^+^ current remodelling, may be more important in increasing the susceptibility of the atria to arrhythmias in HF. However, it is important to note that the data presented here is from a model of end-stage heart failure where contractile dysfunction rather than arrhythmias may be more pathologically relevant.

### 4.4 β-Adrenergic stimulation and restoration of the systolic Ca^2 +^ transient in HF atrial myocytes: potential importance in arrhythmia initiation

In the present study we find that, during β-AR stimulation, the amplitude of the systolic Ca^2 +^ transient in HF atrial cells is indistinguishable from that of control cells. However, neither the rate of decay of the systolic Ca^2 +^ transient (*k*_sys_) nor SR dependent rate of Ca^2 +^ removal (*k*_SR_) is fully restored by β-AR stimulation. Nevertheless, SR Ca^2 +^ content is increased by β-AR stimulation in both control and HF cells and importantly, in HF cells, SR Ca^2 +^ content remains greater than in control cells. It is therefore likely that the combined effect of increasing SR Ca^2 +^ content and *I*_Ca-L_ during β-AR stimulation is responsible for normalizing systolic Ca^2 +^ in HF atrial myocytes. Thus, during β-AR stimulation the increase in excitation contraction coupling gain and fractional release of Ca^2 +^ from the SR [51] facilitate the propagation of the peripheral Ca^2 +^ transient to the cell centre and restore systolic Ca^2 +^ despite the loss of atrial t-tubules in HF [27,52].

Although not addressed specifically in the present study, due to the difficulty in quantitatively measuring changes of SR Ca^2 +^ content from the amplitude of the caffeine evoked Ca^2 +^ transient, should SR Ca^2 +^ content preferentially increase during β-AR stimulation in HF atrial cells following current clamp stimulation (the *in vivo* situation) this would also provide a conducive pathway to DAD formation and act as an important precursor for new onset AF [10]. However, despite the potential effects of changes in SR Ca^2 +^ content following β-AR stimulation during current clamp stimulation, the shorter action potential duration noted in HF atrial cells, via reductions in effective refractory period would also serve to promote arrhythmias although via re-entrant mechanisms.

### 4.5 Molecular correlates of altered cellular Ca^2 +^ homeostasis and β-AR responsiveness in heart failure atrial myocytes

A major observation in the present study is that SERCA activity is reduced in HF atrial cells resulting in reductions to the rate of decay of the systolic Ca^2 +^ transient and SR dependent Ca^2 +^ uptake. Whilst changes in SERCA expression or activity have only modest effects on SR Ca^2 +^ content [53,54] (due to the shallow dependence of SR Ca^2 +^ content on SERCA activity [55]), we noted that SR Ca^2 +^ content was increased in voltage-clamped HF atrial cells. Importantly, the changes in SERCA activity were noted despite the *decrease* in Ca^2 +^ buffering power and therefore, as presented, likely represent an underestimate of the actual decrease in SERCA function. Whilst we were unable to detect changes in SERCA, total PLN, Ser-16 phosphorylated PLN or the ratio of PLN to SERCA, we did observe an increase in Thr-17 phosphorylated PLN, CAMKII, PP1, PP2a and GRK-2 expression. Thus the reported changes in protein expression in HF atria do not readily account for the observed reduction in SERCA activity. In the atria SERCA is also regulated by an additional protein, sarcolipin [56]. However, due to a lack of suitable antibodies we have been unable to investigate whether or not sarcolipin expression alters in HF atrial myocytes and can explain the decrease in SERCA activity we observe.

The failure of β-AR stimulation to completely restore SERCA function and *I*_Ca-L_ in HF atrial cells is consistent with the increased expression of the G-protein receptor kinase GRK-2 [57]. Similar findings have been reported previously in the ventricle in various models of cardiac disease e.g. [5,58]. However, given the effect that reducing *I*_Ca-L_ has on SR Ca^2 +^ content, the increases in GRK-2, PP1 and PP2a may be important in maintaining the increased SR Ca^2 +^ content in HF atrial cells during β-AR stimulation because they attenuate any β-AR mediated increase in *I*_Ca,L_.

### 4.6 Study limitations

We have examined how intracellular Ca^2 +^ homeostasis and the response to catecholamine stimulation are altered in the atria in HF. Whilst the changes in cellular Ca^2 +^ homeostasis and, in particular SR Ca^2 +^ content, may provide a substrate for the generation of Ca^2 +^ dependent arrhythmias we have not established if the threshold SR Ca^2 +^ content is altered in HF, if HF atrial myocytes are more excitable due to concomitant alterations in K^+^ channel expression or function e.g. [15] and whether ultimately AF is more readily induced and maintained in this model of HF. However, in a comparable model of tachypacing induced HF in the dog, an increased susceptibility to, and stability of, AF has been reported [59] and, at the single cell level, spontaneous SR Ca^2 +^ release and DADs were more prevalent in HF atrial myocytes [20]. For the measurements of *I*_Ca-L_ a holding potential of − 40 mV was used and we did not correct for any liquid junction potential artefacts (~ 8 mV).

For the in vivo assessments of cardiac function by ECG and echocardiography animals were gently restrained. As such we cannot exclude the possibility that adrenergic outflow may be influencing our measures of cardiac function in both control and HF animals; and potentially differentially. However, the use of sedation or performing such measurements in anaesthetized animals would also be expected to influence these measurements and again may do so differentially.

In the present study we have used Western blotting to measure the expression of protein phosphatases and kinases and have not investigated their activity directly. However, the expression of protein phosphatases and G-protein receptor kinases has previously been demonstrated to correlate directly with their activity [60,61]. Thus, our observation of increased PP1, PP2a and GRK-2 expression is consistent with the reduced SR Ca^2 +^ uptake rate and impaired *I*_Ca-L_ that are present under basal and β-AR stimulation conditions in HF atrial myocytes. Moreover, we did not determine if changes in PMCA activity occur in this model of HF and therefore whether the correction factor non-NCX mediated Ca^2 +^ extrusion differs between control and HF myocytes. However, even if PMCA activity were to change by 50% this would result in an under or overestimation of SR Ca^2 +^ content by less than 10%.

## 5 Conclusions

In summary we describe a number of changes to atrial Ca^2 +^ homeostasis that occur as a consequence of HF induced by right ventricular tachypacing. Specifically, the decrease in *I*_Ca-L_ and previously reported loss of atrial t-tubules in this model of HF serve to decrease the systolic Ca^2 +^ transient amplitude and increase SR Ca^2 +^ content in voltage-clamped cells. However, the changes in SR Ca^2 +^ content observed in voltage-clamped cells, are insufficient to prevent the smaller rise of systolic Ca^2 +^. Similarly, the observed decrease in cellular Ca^2 +^ buffering capacity is also insufficient to maintain systolic Ca^2 +^ although it does contribute to an increased rate of Ca^2 +^ removal from the cell via the sarcolemmal pathways despite the reduction in NCX current in the atria in heart failure.

## Funding

This work was supported by grants from the British Heart Foundation (FS/12/57, PG/11/16, PG10/89, PG/09/062, FS/09/036, PG/08/078, PG/07/099, the Michael Frazer PhD studentship FS/07/003), European 6th Framework Award (Normacor), University of Manchester Biomedical Research Centre Award (George Lancashire Award) and Wellcome Trust Institutional Strategic Support Fund (ISSF) award (097820).

## Disclosures

None.

## Figures and Tables

**Table 1:** Modelling the effects of Ca^2 +^ handling alterations in heart failure on systolic Ca^2 +^. Summary of how HF affects the total Ca^2 +^ economy of the cell. *^ns^*, not significant; *, *P* < 0.05; ^#^, *P* < 0.01 and ^§^, *P* < 0.001. The following equations were used to calculate the indicated parameters: Ca^2 +^ buffering power (β) = Δ Ca^2 +^ total/Δ [Ca^2 +^]_i_; Total Ca^2 +^ transient = β ∗ Δ [Ca^2 +^]_i_; systolic Δ SR Ca^2 +^ content = SR Ca^2 +^ content − (Total Ca^2 +^ − ʃ*I*_Ca-L_); SR fractional release = Systolic Δ SR Ca^2 +^/SR Ca^2 +^ content; EC coupling gain = Systolic Δ SR Ca^2 +^/ʃ*I*_Ca-L_.

	Control Atrial cell (*n* = 43)	Heart failure Atrial cell (*n* = 21)
Δ systolic [Ca^2 +^]_i_ (nmol/l)	83 ± 6	60 ± 6^#^
Ca^2 +^ buffering power (β)	565 ± 41	318 ± 29^§^
Total Ca^2 +^ transient (μmol/l)	46.9 ± 4.8	19.1 ± 2.6^§^
Integrated *I*_Ca-L_ (μmol/l)	0.74 ± 0.06	0.86 ± 0.09*^ns^*
SR Ca^2 +^ content (μmol/l)	83.9 ± 4.5	100.1 ± 6.7^⁎^
Δ SR Ca^2 +^ systole (μmol/l)	46.2 ± 4.8	18.2 ± 2.6^§^
SR fractional release	0.55 ± 0.06	0.18 ± 0.03^§^
EC coupling gain	62.4 ± 8.2	21.2 ± 3.8^§^
