# Supplementary material for: Perturbed atrial calcium handling in an ovine model of heart failure: Potential roles for reductions in the L-type calcium current
Source: J Mol Cell Cardiol. 2015 Feb;79:169–79. doi: 10.1016/j.yjmcc.2014.11.017 (PMC4312356; doi:10.1016/j.yjmcc.2014.11.017)
Supplement: Supplementary file 1 — Supplementary material. [file mmc1.pdf]

**SUPPLEMENTARY DATA – Clarke *et al.***

PERTURBED ATRIAL CALCIUM HANDLING IN AN OVINE MODEL OF HEART FAILURE: POTENTIAL ROLES FOR REDUCTIONS IN THE L-TYPE CALCIUM CURRENT

## *1. Supplementary Methods:*

### *1.1. Induction of heart failure by right ventricular tachypacing*

Animals were randomly assigned to control or HF groups and maintained in group housing with 12:12 light:dark cycling and fed ad libitum hay. HF was induced in female Welsh sheep by transvenous right ventricular tachypacing as described in detail previously [1,2]. Following induction and maintenance of anaesthesia (isoflurane, 2-4% in oxygen) and provision of analgesia (meloxicam, 0.5 mg/kg sc.) and antibiosis (enrofloxacin 2.5 mg/kg sc.) a single bipolar active fixation cardiac pacing lead (Medtronic CapSure) was fixed at the right ventricular endocardial apex under fluoroscopic guidance (Philips BV Pulsera). Lead impedance and pacing threshold were then determined and considered adequate if impedance and pacing threshold were less that 1 kΩ and 2 volts respectively. The lead was then connected to cardiac pacemaker (Medtronic Kappa) and the pacemaker body buried subcutaneously in the right lateral cervical region. Animals were then allowed to recover from the surgical procedure for 7 – 10 days before rapid ventricular pacing was commenced (210 beats per minute) to induce HF.

### *1.2. Cellular electrophysiology and surface area to volume ratio calculations*

Following onset of clinical signs of HF (lethargy, dyspnoea, cachexia) [1,2] animals were killed (200 mg/kg pentobarbitone and 10,000 units heaprin iv.) and the heart rapidly removed and single left atrial myocytes were isolated from the left atrial appendage using a collagenase and protease digestion technique as described in detail previously [1].

Changes in intracellular $Ca^{2+}$ concentration ($[Ca^{2+}]_i$) were monitored using the acetoxymethyl ester of either Fluo-5F (5 μmol/l) or Fura-2 (5 μmol/l). Following indicator loading cells were allowed to de-esterify for at least 30 mins at room temperature before experimental use.

An *in vitro* calibration was used to convert Fura-2 emission ratio ($F_{340}/F_{380}$) to changes in $[Ca^{2+}]_i$ and obtain a value for diastolic $[Ca^{2+}]_i$ [3]. We then used the diastolic $[Ca^{2+}]_i$ to calibrate the Fluo-5F signals as described originally by Cheng *et al* [4] using equation 1:

$[Ca^{2+}]_i = KR/[(K/[Ca^{2+}]_{rest} + 1) - R]$ equation 1.

Where K = dissociation constant of Fluo-5f for $Ca^{2+}$ (1035 nmol/l [5]), R is the pseudo-ratio ($R/R_{rest}$) and $[Ca^{2+}]_{rest}$ the diastolic $[Ca^{2+}]_i$ determined using the Fura-2 calibration method above.

For voltage clamp studies using the perforated patch technique, patch pipettes (2-3 MΩ) were filled with (in mmol/l) $KCH_3O_3S$, 125; KCl, 20; NaCl, 10; HEPES, 10, $MgCl_2$, 5; pH 7.2 with KOH. Series resistance (39.0 ± 3.1 MΩ) errors were overcome using the 'switch-clamp' facility of the Axoclamp-2B amplifier (Molecular Devices, UK).

To calculate the surface area to volume ratio cells were loaded with calcein-AM (20 μmol/l) and cell volume calculated from the rendered 3 dimensional image stack obtained on a Leica SP2 confocal microscope as described previously for atrial [6] and ventricular myocytes [7]. Calcein loaded cells were simultaneously patch-clamped and cell capacitance used as an indicator of cell surface area (10 mV hyperpolarizing step applied from a holding potential of -40 mV). All integrated $Ca^{2+}$ flux measurements (SR $Ca^{2+}$ content, total $Ca^{2+}$ and L-type $Ca^{2+}$ current) are expressed relative to the *total* cell-volume obtained from the cell-type specific surface area : volume ratios.

SR $Ca^{2+}$ content was quantified by integrating the NCX current evoked by discharging the SR $Ca^{2+}$ store on rapid application of caffeine (10 mmol/l) [8]. To correct for $Ca^{2+}$ removal by the electro-neutral plasmalemmal $Ca^{2+}$-ATPase a separate series of filed stimulation experiments were performed and NCX mediated $Ca^{2+}$ efflux inhibited with 10 mM $NiCl_2$. The decay of the subsequent caffeine evoked rise of $Ca^{2+}$ was fitted with a single exponential to determine the NCX dependent and independent contributions to $Ca^{2+}$ removal as described in detail previously [6,9,10]. A correction factor of 1.2 was calculated.

The $Ca^{2+}$ buffering capacity of atrial cells was also determined from the caffeine evoked rises of $[Ca^{2+}]_i$ and integrated NCX current. Briefly the integrated NCX current provides a measure of *total* $Ca^{2+}$ and plotting this as a function of the *free* intracellular $Ca^{2+}$ measured with the fluorescent indicator. This data has then been fit with a linear regression and the slope of the regression used as an index of $Ca^{2+}$ buffering capacity. [11].

Cells were perfused (at 37 $^{o}C$) with a standard solution containing (in mmol/l): NaCl, 140; glucose, 10; HEPES, 10; KCl, 4; $CaCl_2$, 1.8; $MgCl_2$, 1; probenecid, 2; pH 7.4 with NaOH. Contaminating $K^+$ currents were blocked (in voltage-clamp experiments) as required by inclusion of 4-aminopyridine (5 mmol/l) and $BaCl_2$ (0.1 mmol/l) in the superfusate. The $Ca^{2+}$ activated $Cl^-$current was blocked with 4,4'-diisothiocyanatostilbene-2,2'-disulphonic acid disodium salt (0.1 mmol/l).

*1.3. Immunoblotting details*

| Primary antibody | Protein loaded (µg), primary antibody concentration and time | Blocking conditions | Secondary antibody concentration and time |
|---|---|---|---|
| SERCA2a (sc73022, Santa Cruz) | 5µg, 1:5000, overnight 4°C | Superblock (Thermo Scientific), 1 hour RT | 1:5000, 1 hour RT |
| Phospholamban (A010-14, Badrilla) | 5µg, 1:10,000, overnight 4°C | Superblock (Thermo Scientific), 1 hour RT | 1:5000, 1 hour RT |
| Calsequestrin (PA1-913, Affinity Bioreagents) | 10µg, 1:2500, overnight 4°C | Superblock (Thermo Scientific), 1 hour RT | 1:25,000, 1 hour RT |
| Ser16 PLN (A010-12, Badrilla) | 20µg, 1:5000, overnight 4°C | 5% Blotto (Santa Cruz), 1 hour RT | 1:10,000, 1 hour RT |
| Thr17 PLN (A010-13, Badrilla) | 20µg, 1:5000, overnight 4°C | 5% Blotto (Santa Cruz), 1 hour RT | 1:10,000, 1 hour RT |
| CAMKIIδ (sc5392, Santa Cruz) | 10µg, 1:2500, overnight 4°C | SEABLOCK (Thermo Scientific), 75mins RT | 1:5000, 1 hour RT |
| PP1 (sc7482, Santa Cruz) | 20µg, 1:2000, overnight 4°C | Superblock (Thermo Scientific), 1 hour RT | 1:5000, 1 hour RT |
| PP2a (sc14020, Santa Cruz) | 20µg, 1:2000, overnight 4°C | 5% Blotto (Santa Cruz), 75mins RT | 1:500, 90mins RT |
| GRK-2 (sc562, Santa Cruz) | 20µg, 1:1000, overnight 4°C | 5% Blotto (Santa Cruz), 1 hour RT | 1:500, 90mins RT |
| RyR (ab2827, Abcam) | 10µg, 1:1000, 2 hours RT | Superblock (Thermo Scientific), 45mins RT | 1:5000, 1 hour RT |
| Ser2808 RyR (A010-10, Badrilla) | 10µg, 1:5000, 2 hours RT | Superblock (Thermo Scientific), 45mins RT | 1:10,000, 1 hour RT |
| Ser2814 RyR (A010-31, Badrilla) | 10µg, 1:2500, 2 hours RT | Superblock (Thermo Scientific), 1 hour RT | 1:10,000, 1 hour RT |

*Table 1. Details of protein loading, antibody source, blotting, blocking and detection conditions used for Western blotting.* All secondary antibodies were horseradish peroxidase conjugated and raised against the appropriate species.

## *2. Supplementary Data:*

### *2.1. Cellular hypertrophy and surface area to volume determination*

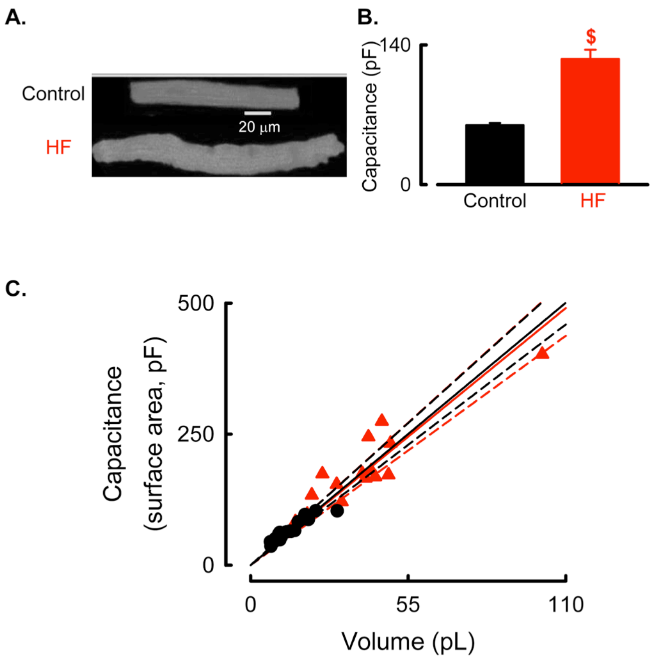


*Figure SI. Cellular hypertrophy and unaltered surface area to volume relationship in heart failure atrial myocytes*

**A.** Representative planar views of single atrial cells loaded with the fluorescent indicator calcein-AM. **B.** Mean data summarizing cellular capacitance changes in heart failure. **C.** Capacitance (surface area) measurements as a function of cell volume in control (black circles) and heart failure (red triangles) atrial myocytes. Both sets of data have been fitted with a linear regression passing through the origin (solid lines) and 95 % confidence intervals are shown (broken lines). $^{\$}$, $P < 0.001$.

*2.2. 'Housekeeping' proteins are altered in heart failure*

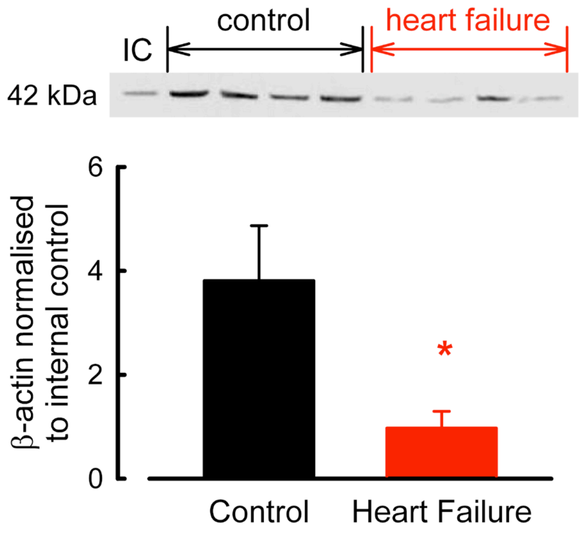


*Figure SII. The housekeeping protein β-actin is reduced in heart failure.*

Representative Western blot showing β-actin immunoreactivity for control and heart failure atrial tissue samples (upper panel) and summary data (lower panel). IC, internal control; *, $P < 0.05$.

*2.3. Heart failure mediated changes in action potential duration do not cause the smaller systolic $Ca^{2+}$ transient or alter SR $Ca^{2+}$ content.*

We used the action potential clamp technique to determine if the shortening of action potential duration noted in HF atrial myocytes was responsible for the smaller systolic $Ca^{2+}$ transient in HF atrial myocytes. Cells isolated from control sheep were voltage-clamped using the perforated patch-clamp technique with switch clamping and the averaged control or HF action potential (main manuscript, fig 1) used as the command waveform applied to the cells. Changes in $[Ca^{2+}]_i$ were measured using Fluo-5F and calibrated as described above. Caffeine (10 mmol/l) was applied to quantify SR $Ca^{2+}$ content. Using the action potential clamp technique we determined that changes in action potential duration were not responsible for either the smaller systolic $Ca^{2+}$ transient nor are they responsible for any change of SR $Ca^{2+}$ content in control myocytes.

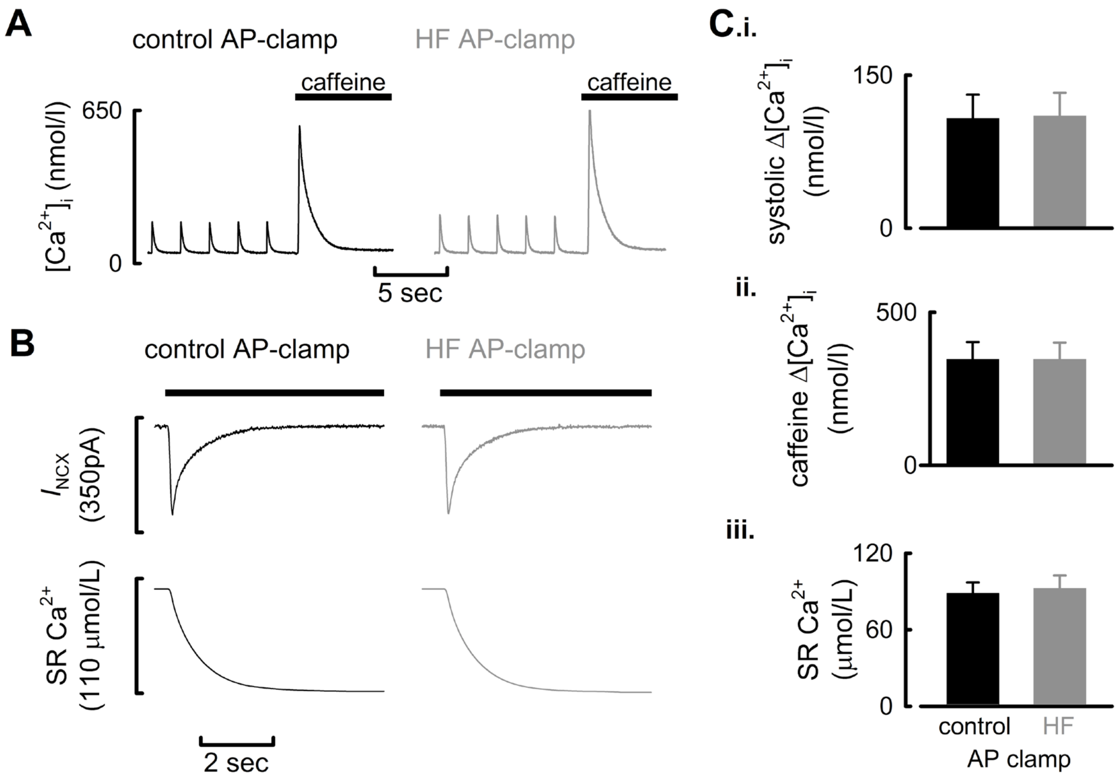

*Figure S.III. Action potential duration changes in heart failure are not responsible for changes in systolic $Ca^{2+}$.*

**A.** Representative experimental time course showing systolic $Ca^{2+}$ and caffeine-evokked $Ca^{2+}$ transients in response to control or HF action potential command waveforms as indicated. **B.** Quantification of SR $Ca^{2+}$ content following control or heart failure action potential command waveforms. **C.** Summary data for (i) $Ca^{2+}$ transient amplitude, (ii) caffeine-evoked transient amplitude and, (iii) SR $Ca^{2+}$ content determined from the integral of the NCX current evoked on caffeine application.

*2.4. Decreased $Ca^{2+}$ buffering power in heart failure accelerates sarcolemmal mediated $Ca^{2+}$ extrusion.*

Here we sought to determine the role that decreased $Ca^{2+}$ buffering in HF explains the paradoxical decrease in NCX current (fig 3, main manuscript) yet increased rate of sarvcolemmal mediated $Ca^{2+}$ extrusion ($k_{caff}$, fig 5 main manuscript). Changes in $[Ca^{2+}]_i$ were converted to *total* $Ca^{2+}$ ($Ca_T$) and the relationship between the rate of fall of $Ca_T$ and $[Ca^{2+}]_i$ (free $Ca^{2+}$) examined during systolic and caffeine evoked rises of $[Ca^{2+}]_i$ for representative control and HF atrial myocytes. It is clear that the rate of fall of $Ca_T$ during the systolic $Ca^{2+}$ transient is fastest in the control cell whilst that for the caffeine evoked rise of $[Ca^{2+}]_i$ is fastest in the HF cell. We then examined the relationship between the sarcolemmal dependent rate of $Ca^{2+}$ extrusion ($k_{caff}$) and cellular $Ca^{2+}$ buffering power and found an inverse correlation exists ($P = 0.01$) indicating that the decrease in $Ca^{2+}$ buffering capacity is potentially the mechanism whereby $k_{caff}$ is increased in HF despite a reduced NCX current density.

A.

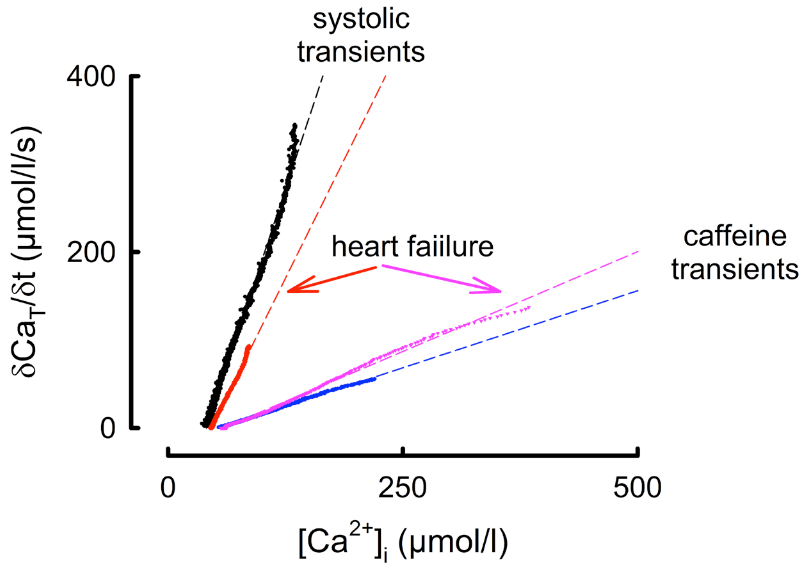


B.

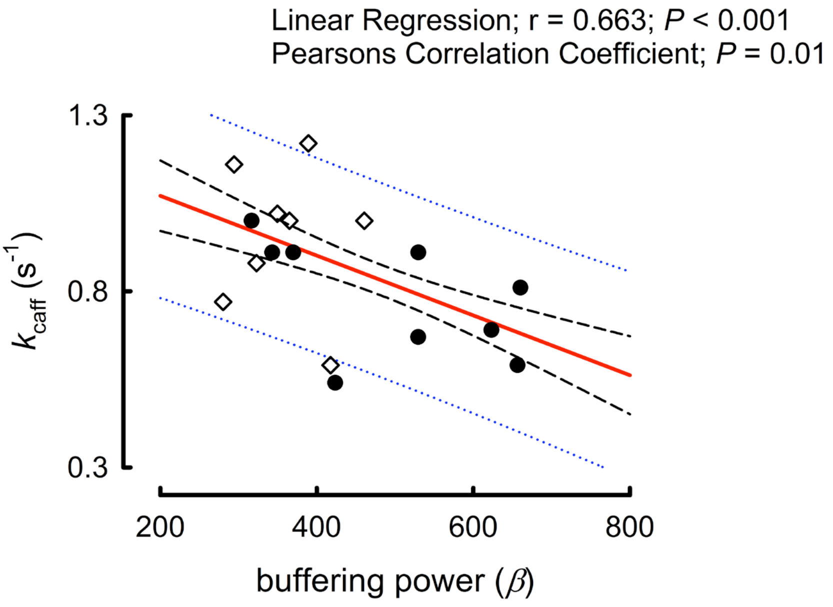


*Figure S.IV. Reduced $Ca^{2+}$ buffering power in heart failure*

**A.** Representative rates of fall of total $Ca^{2+}$ ($\delta Ca_T/\delta t$) as a function of $[Ca^{2+}]_i$ (free $Ca^{2+}$) for systolic and caffeine evoked rises of $[Ca^{2+}]_i$ for the cell types as indicated. **B.** Inverse correlation between the sarcolemmal mediated rate of $Ca^{2+}$ extrusion ($k_{caff}$) and cellular $Ca^{2+}$ buffering power. The solid symbols represent control hearts (solid symbols) and

the open symbols HF (open symbols)  hearts.  The solid red line through the data is a best fit linear regression, the dotted line 95 % confidence intervals and the dotted blue lines the prediction limits.

### *2.5. Protein expression changes in heart failure*

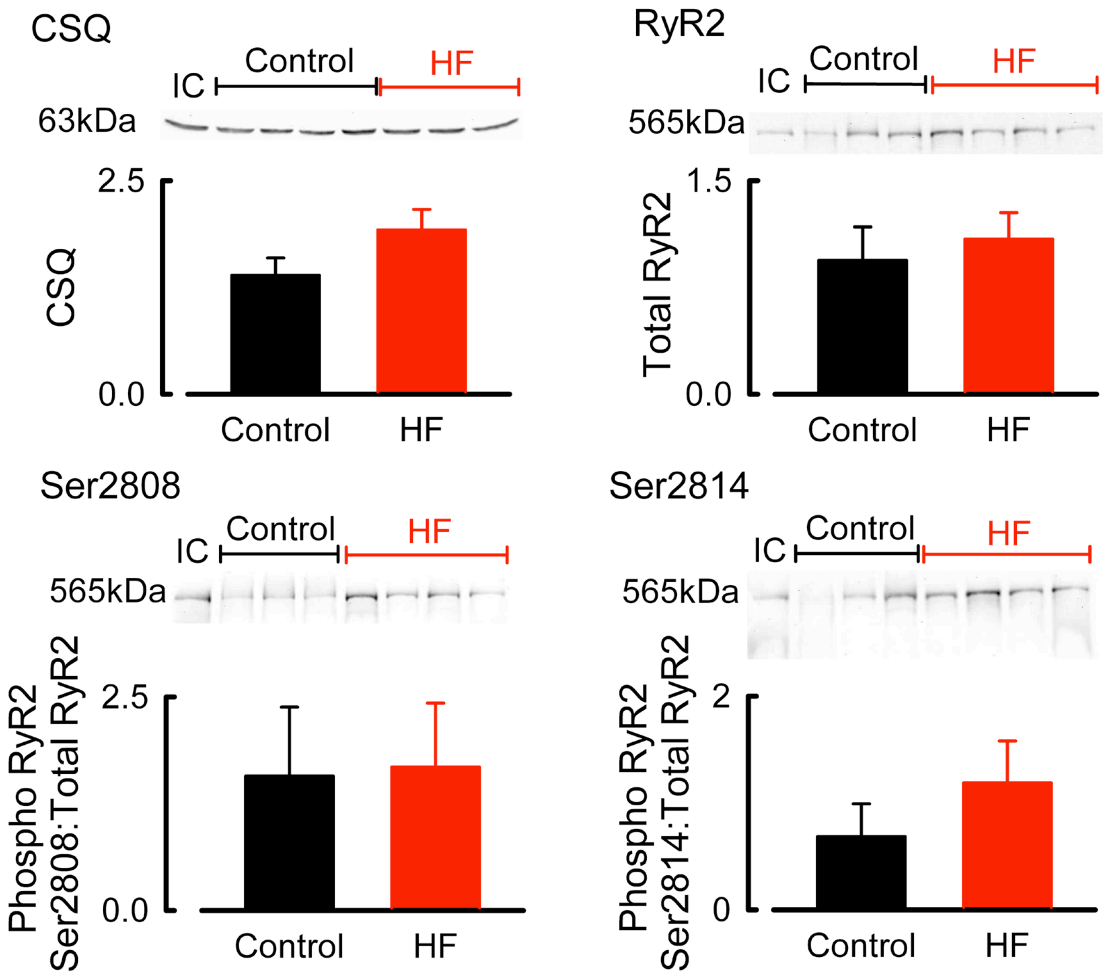


*Figure S.V.  Unaltered protein expression in heart failure.*

Data shown for calsequestrin (CSQ), the ryanodine receptor (RyR2) and RyR2 phosphorylation status at Ser 2808 and Ser 2814.  Panels show representative immunoblots (upper) and summary data (lower). IC, internal control.

*2.6. G-protein subunit expression in heart failure*

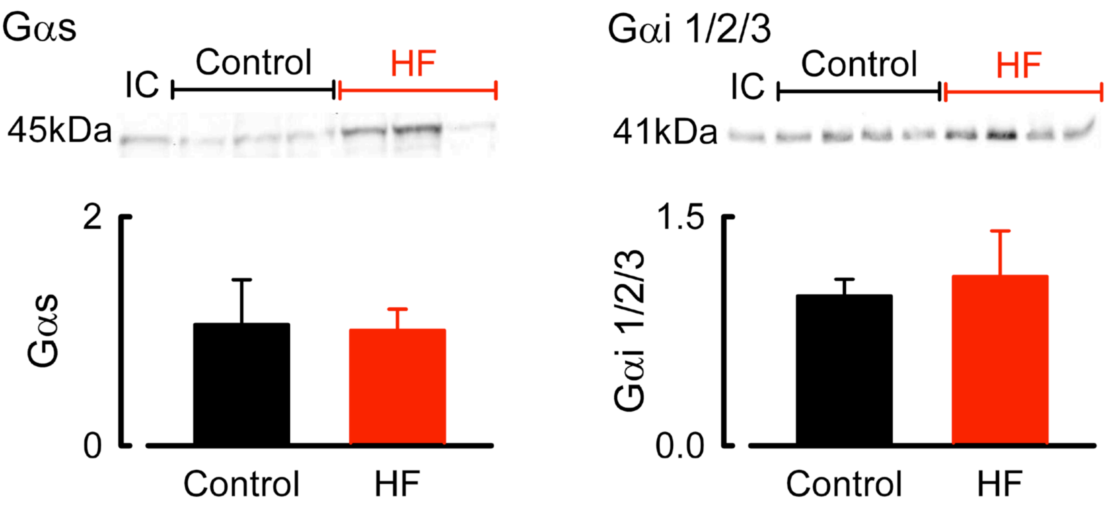


*Figure S.VI. G-protein α subunit expression is unaltered in heart failure atrial tissues*

Representative immunoblots (upper panels) for $G_{\alpha s}$ (left) and $G_{\alpha i,1,2,3}$ (right) subunit expression and summary data (lower panels). IC, internal control.

*2.5. Right ventricular tachypacing does not increase atrial rate*

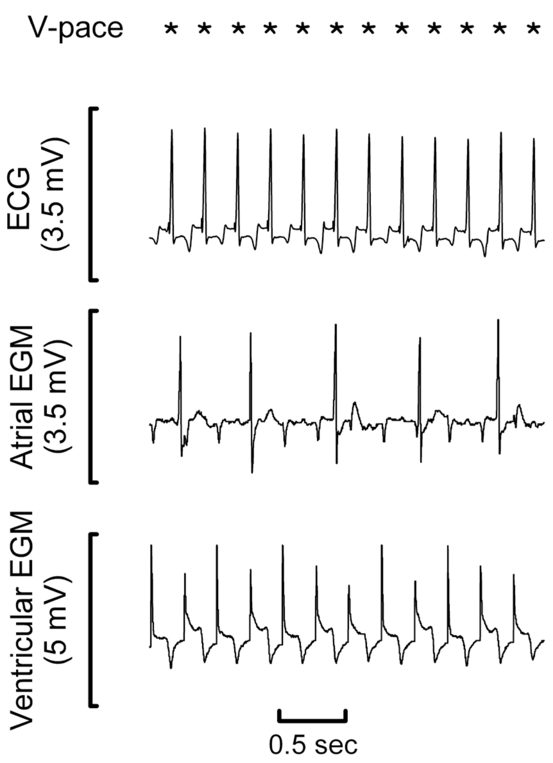


*Figure SV. Ventricular pacing does not lead to atrial capture.*

A sub-group of sheep were instrumented with dual chamber pacemakers. In addition to the right ventricular apical pacing lead a second pacing lead (used to sense atrial activity) was fixed to the right atrial appendage. Panels show the surface electrocardiogram (upper), atrial electrogram (middle) and ventricular electrogram (lower). During ventricular tachypacing (V-pace, *) at 210 beats per minute the surface ECG and ventricular electrogram show ventricular capture on each stimulus whereas the rate of atrial depolarisation remains unaffected on the atrial electrogram.

## References


[1] Dibb KM, Clarke JD, Horn MA, Richards MA, Graham HK, Eisner DA, et al. Characterization of an extensive transverse tubular network in sheep atrial myocytes and its depletion in heart failure. Circ Heart Fail 2009;2:482-9.

[2] Briston SJ, Caldwell JL, Horn MA, Clarke JD, Richards MA, Greensmith DJ, et al. Impaired β-adrenergic responsiveness accentuates dysfunctional excitation contraction coupling in an ovine model of tachypacing induced heart failure. J Physiol 2011;589:1367-1382.

[3] Grynkiewicz G, Poenie M, Tsien RY. A new generation of ca2+ indicators with greatly improved fluorescence properties. J Biol Chem 1985;260:3440-50.

[4] Cheng H, Lederer WJ, Cannell MB. Calcium sparks: Elementary events underlying excitation-contraction coupling in heart muscle. Science 1993;262:740-4.

[5] Loughrey CM, MacEachern KE, Neary P, Smith GL. The relationship between intracellular [ca(2+)] and ca(2+) wave characteristics in permeabilised cardiomyocytes from the rabbit. J Physiol 2002;543:859-70.

[6] Walden AP, Dibb KM, Trafford AW. Differences in intracellular calcium homeostasis between atrial and ventricular myocytes. J Mol Cell Cardiol 2009;46:463-73.

[7] Satoh H, Delbridge LM, Blatter LA, Bers DM. Surface:Volume relationship in cardiac myocytes studied with confocal microscopy and membrane capacitance measurements: Species-dependence and developmental effects. Biophys J 1996;70:1494-504.

[8] Varro A, Negretti N, Hester SB, Eisner DA. An estimate of the calcium content of the sarcoplasmic reticulum in rat ventricular myocytes. Pflugers Arch 1993;423:158-60.

[9] Díaz ME, Graham HK, Trafford AW. Enhanced sarcolemmal $ca2^{+}$ efflux reduces sarcoplasmic reticulum $ca2^{+}$ content and systolic $ca2^{+}$ in cardiac hypertrophy. Cardiovasc Res 2004;62:538-47.

[10] Dibb KM, Rueckschloss U, Eisner DA, Isenberg G, Trafford AW. Mechanisms underlying enhanced cardiac excitation contraction coupling observed in the senescent sheep myocardium. J Mol Cell Cardiol 2004;37:1171-81.

[11] Trafford AW, Díaz ME, Eisner DA. A novel, rapid and reversible method to measure ca buffering and time-course of total sarcoplasmic reticulum ca content in cardiac ventricular myocytes. Pflugers Arch 1999;437:501-3.
